# Fatty Acid Binding Protein 4 Deficiency Protects against Oxygen-Induced Retinopathy in Mice

**DOI:** 10.1371/journal.pone.0096253

**Published:** 2014-05-06

**Authors:** Magali Saint-Geniez, Elisa Ghelfi, Xiaoliang Liang, Chenwei Yu, Carrie Spencer, Stephanie Abend, Gokhan Hotamisligil, Sule Cataltepe

**Affiliations:** 1 Schepens Eye Research Institute, Massachusetts Eye and Ear, Department of Ophthalmology, Harvard Medical School, Boston, Massachusetts, United States of America; 2 Department of Neonatology, Brigham and Women's Hospital and Harvard Medical School, Boston, Massachusetts, United States of America; 3 Department of Genetics and Complex Diseases, Harvard School of Public Health, Boston, Massachusetts, United States of America; University of Sydney, Australia

## Abstract

Retinopathy of prematurity (ROP) is a leading cause of blindness in children worldwide due to increasing survival rates of premature infants. Initial suppression, followed by increased production of the retinal vascular endothelial growth factor-A (VEGF) expression are key events that trigger the pathological neovascularization in ROP. Fatty acid binding protein 4 (FABP4) is an intracellular lipid chaperone that is induced by VEGF in a subset of endothelial cells. FABP4 exhibits a pro-angiogenic function in cultured endothelial cells and in airway microvasculature, but whether it plays a role in modulation of retinal angiogenesis is not known. We hypothesized that FABP4 deficiency could ameliorate pathological retinal vascularization and investigated this hypothesis using a well-characterized mouse model of oxygen-induced retinopathy (OIR). We found that FABP4 was not expressed in retinal vessels, but was present in resident macrophages/microglial cells and endothelial cells of the hyaloid vasculature in the immature retina. While FABP4 expression was not required for normal development of retinal vessels, FABP4 expression was upregulated and localized to neovascular tufts in OIR. FABP4^−/−^ mice demonstrated a significant decrease in neovessel formation as well as a significant improvement in physiological revascularization of the avascular retinal tissues. These alterations in retinal vasculature were accompanied by reduced endothelial cell proliferation, but no effect on apoptosis or macrophage/microglia recruitment. FABP4^−/−^ OIR samples demonstrated decreased expression of genes involved in angiogenesis, such as Placental Growth Factor, and angiopoietin 2. Collectively, our findings suggest FABP4 as a potential target of pathologic retinal angiogenesis in proliferative retinopathies.

## Introduction

Retinopathy of prematurity (ROP) has become a leading cause of blindness in children worldwide due to increasing survival rates of premature infants[1]. Severe ROP affects 30–40% of infants born between 23–26 weeks gestational age[2]. Although the current laser ablation treatment reduces the incidence of blindness in infants with late-stage ROP, it can lead to other problems, such as development of abnormal retinal structure, strabismus, glaucoma and nystagmus[3]–[5].

ROP is a biphasic disease that is associated with abnormal retinal vascular development[1], [6]. The first phase of ROP is characterized by vaso-obliteration and cessation of normal blood vessel growth in the retina. Suppression of retinal vascular endothelial growth factor (VEGF)-A expression by relative hyperoxia is one of the key events that triggers the pathology associated with phase 1 ROP, which lasts until approximately 30–32 weeks corrected gestational age. In phase 2 or the proliferative phase of ROP, increased metabolic activity of the maturing retina leads to relative hypoxia and increased production of VEGF-A as well as other angiogenic factors, such as insulin-like growth factor 1 (IGF-1) and erythropoietin (EPO). The key role of VEGF-A in the pathogenesis of ROP has been underscored in a recent clinical trial, where intravitreal administration of a VEGF-A-blocking antibody, bevacizumab, was found to be superior to laser therapy in infants with severe ROP[7], [8]. However, both pre-clinical and clinical studies have well documented the critical homeostatic and trophic function of VEGF-A not only on the vasculature but also on neurons and epithelial cells[9], [10]. As intravitreal bevacizumab administration decreases serum levels of VEGF-A, there are significant concerns regarding the potential systemic side effects and long-term outcomes associated with this treatment[11]–[13].

Fatty acid binding protein 4 (FABP4) is a small molecular weight intracellular lipid chaperone, which plays an important role in regulation of lipid and glucose homeostasis as well as inflammation through its actions in adipocytes and macrophages[14]–[18]. We have recently reported that FABP4 is also expressed in a subset of endothelial cells (EC), where its expression is positively regulated by the VEGF/VEGFR2 and mTORC1 pathways[19]–[21]. In contrast to pan-endothelial cell markers, such as CD31, FABP4 expression is restricted to microvascular and small vascular ECs, which are actively involved in angiogenic responses. Consistent with this notion, deficiency of EC-FABP4 leads to attenuation of angiogenic responses, such as cell proliferation, migration, survival, and morphogenesis through modulation of several pathways, including eNOS and SCF/c-kit signaling[19], [22]. These findings were recently corroborated in a transgenic mouse model, where FABP4 knock-out mice were protected from VEGF-induced airway angiogenesis and inflammation due to the lack of EC-FABP4[23]. Furthermore studies in human subjects with glioblastoma, the most malignant primary brain tumor in adults associated with robust angiogenic activity, revealed FABP4 expression in a subset of ECs, which was not detected in control specimens[21].

Taken together, these studies have suggested that FABP4 might enhance pathological angiogenesis, which plays a role in the pathogenesis of ROP. Thus we hypothesized that genetic deficiency of FABP4 could ameliorate pathological retinal neovascularization that occurs in ROP and investigated this hypothesis using a well-characterized mouse model of oxygen-induced retinopathy (OIR)[24].

## Results

### FABP4 is induced during retinal development but is not required for normal vascular development

**Figure 1:**
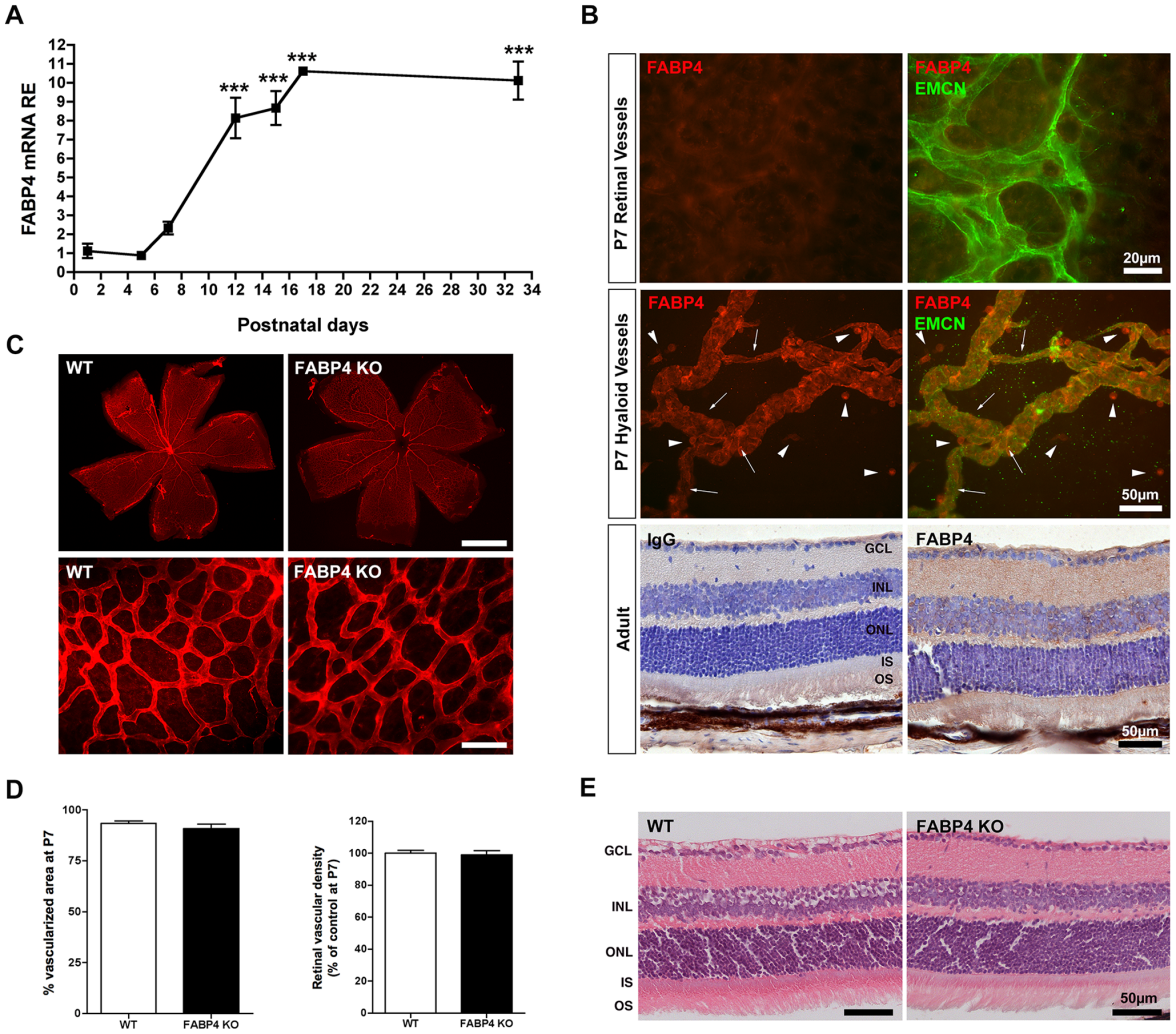
FABP4 expression and function during normal retinal development. (A) qPCR quantification of FABP4 mRNA in total retinas from P1 to P33 WT mice. Results are expressed as RE (relative expression) normalized to housekeeping genes (mean ± SEM, n = 3–4). FABP4 is significantly induced during the postnatal stage of retinal development. (B) FABP4 distribution was determined on retina flat-mounts and paraffin sections. At P7, FABP4 expression (red) was not observed in the inner retinal vessels stained by the pan-EC marker EMCN (green). FABP4 was strongly detected in the vitreal macrophages (arrowheads) and hyaloid endothelial cells (arrows). Paraffin sections of adult retina demonstrate faint, but uniform expression of FABP4 in all retinal layers. (C) WT and FABP4^−/−^ P7 flat-mount retinas were immunostained with Collagen IV to identify the vascular network. (D) Vascular coverage and density was similar in WT and FABP4^−/−^ animals indicating that FABP4 expression is not required for normal retinal vascularization. (E) Hematoxylin and eosin stained sagittal retinal sections of adult WT and FABP4^−/−^ mice showed no morphological anomalies or degeneration in FABP4^−/−^ animals. gcl: ganglion cell layer; ipl: inner plexiform layer; inl: inner nuclear layer; onl: outer nuclear layer; os: outer segment.

To investigate the role of FABP4 in retinal vasculature, we first characterized the relative expression of FABP4 during the postnatal developmental stages of the mouse retina. As shown in Figure 1A, FABP4 is strongly induced in retinal tissues starting at P5 and peaking at P17, and its level plateaus in mature retinas at P33. Using immunostaining on retinal flat-mounts and sections, we did not detect FABP4 expression in developing or mature retinal vessels, but identified robust FABP4 expression in resident macrophages/microglias and EC of the hyaloid vasculature (Figure 1B). As expected, FABP4 was also detected in other non-neuronal ocular and periocular vascular beds such as the limbal vessels and ocular muscles capillaries (Figure S1). In the mature retina, following the complete regression of the hyaloid vessels, faint and ubiquitous FABP4 immunoreactivity was detected throughout the retinal layers. Since FABP4 induction reaches its maximum levels at the time of completion and maturation of the inner retinal vasculature, FABP4 could play a role during normal retinal vascularization. To investigate this possibility, we used P7 flat-mount retinas stained for the blood vessel basal membrane marker, collagen IV, and found a similar percentage of vascularized area in WT (93.39±1.24, n = 4) and FABP4^−/−^ (90.83±2.32, n = 5) animals (Figure 1C). Similarly, quantification of vascular density at P7 did not reveal any difference between WT (37.87±0.70, n = 4) and FABP4^−/−^ (37.49±0.96, n = 6) retinas (Figure 1C). These results demonstrate that FABP4 expression is not required for normal development of retinal vessels. Furthermore, histological analysis of adult FABP4^−/−^ retina showed no signs of abnormal morphology or thinning (Figure 1D), thus also demonstrating that FABP4 is not required for development and survival of the neuroretina.

### FABP4 is specifically induced in neovascular tufts in OIR

**Figure 2:**
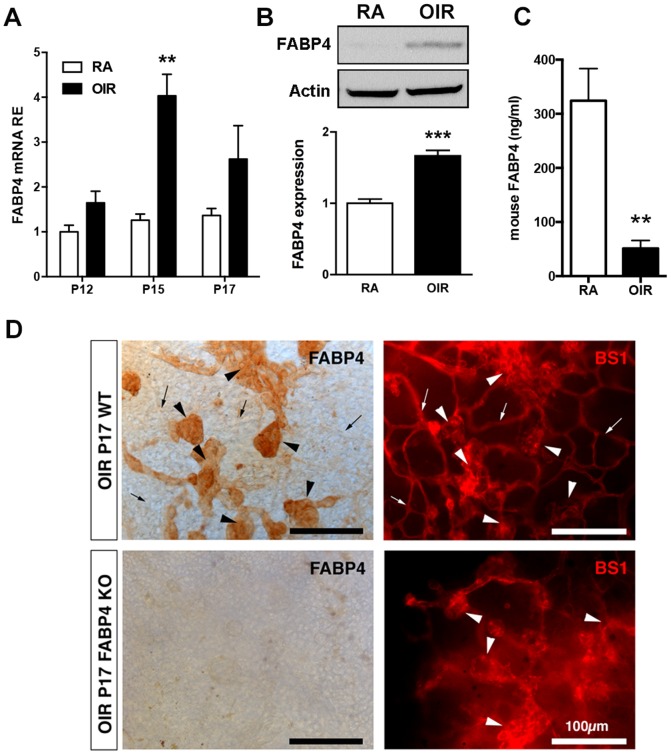
Specific induction of FABP4 in neovessels during OIR. (A) qPCR quantification of FABP4 mRNA in total retinas from P12 to P17 in control (RA = room air) and OIR WT mice. Results are expressed as RE (relative expression) normalized to housekeeping genes (mean ± SEM, n = 3–4). FABP4 mRNA is significantly induced during the angiogenic phase of OIR. (B) Quantification of FABP4 protein expression in P17 RA control and OIR retinas by immunoblot and densitometry analysis (mean ± SEM, n  =  6) confirmed up-regulation of FABP4 expression during pathological neovascularization. (C) Quantification of circulating FABP4 demonstrated a significant decrease of serum FABP4 levels in OIR P17 WT mice compared to control room air (RA). Results are presented as mean ± SEM based on n = 3–4 individual animals. (D) FABP4 distribution was determined on P17 retina flat-mounts from OIR animals co-stained with the pan-endothelial marker BS1. FABP4 is expressed in neovascular tufts (arrowheads) and absent from adjacent normal blood vessels (arrows). Specificity of the FABP4 staining is confirmed using FABP4^−/−^ retina. Scale bar is 100 µm.

**Figure 3:**
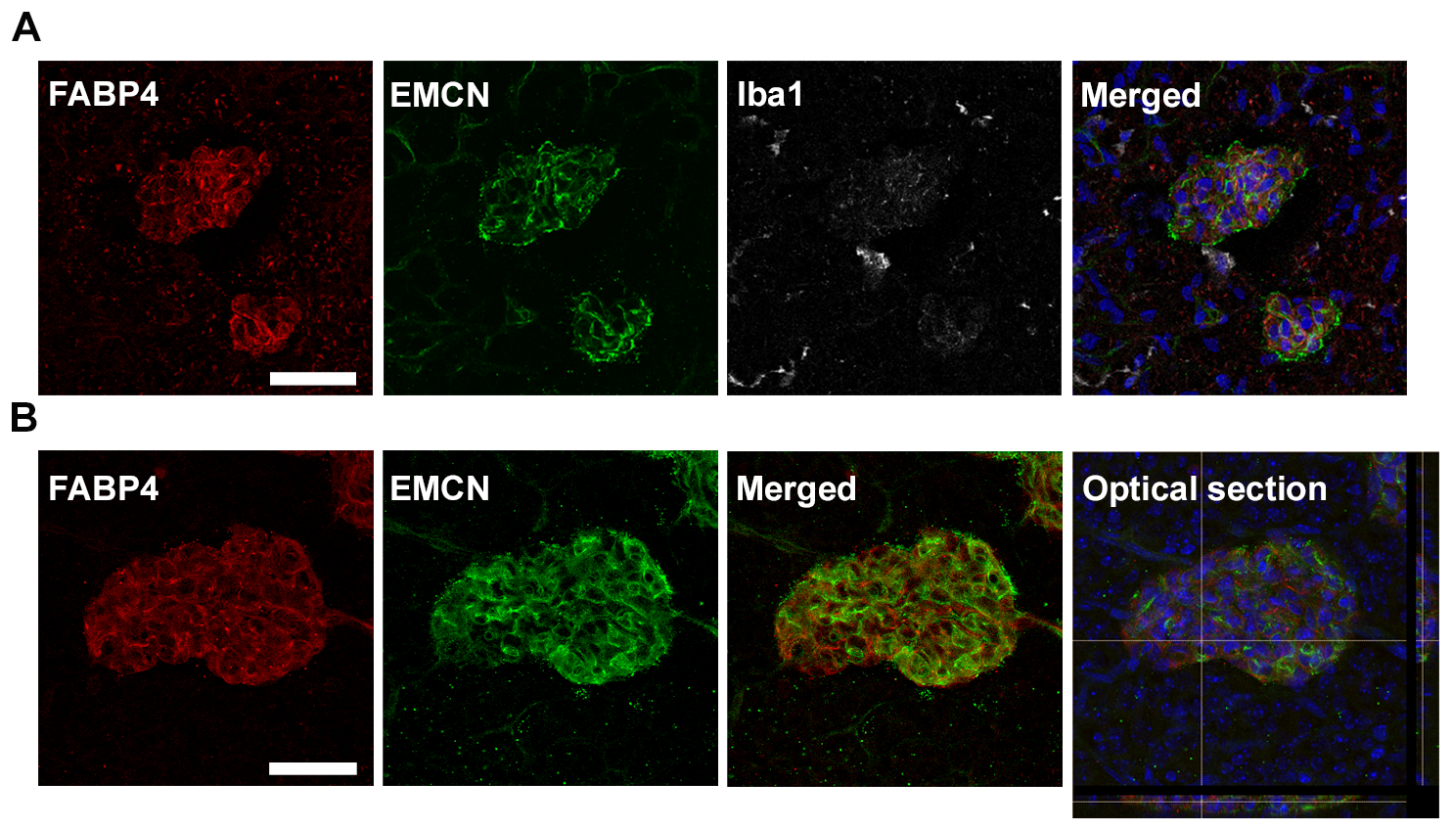
Specific localization of FABP4 in neovascular endothelial cells. (A) Confocal imaging of neovascular tufts in P17 WT OIR retina flat-mount following immunostaining for FABP4 (red), EC-specific marker EMCN (green), activated microglia-specific marker Iba1 (white) and DAPI (blue). FABP4 is strongly associated with the neovascular tufts and absent from the normal vasculature. FABP4 was not detected in the Iba1- positive cells but co-localized with EMCN-positive cells. (B) Immunostaining of FABP4 (red) EMCN (green) and DAPI (blue) in a large neovascular structure followed by confocal sectioning confirmed FABP4 localization to ECs of pathological neovessels. Scale bar is 50 µm.

Our previous studies have demonstrated that FABP4 expression in EC is strongly upregulated by VEGF-A, a key mediator of pathological neovascularization in OIR. Therefore we next determined whether FABP4 expression was upregulated in the OIR model, where VEGF is dramatically induced by the hypoxic retinal tissue. Indeed, we observed a rapid and significant increase in FABP4 mRNA expression in the hypoxic retina at the stage of most active angiogenic response (P15–P17) (Figure 2A). Western-blot analysis confirmed induction of FABP4 at the protein level in OIR (Figure 2B). Contrary to the increased FABP4 expression observed in the retinal tissue, circulating FABP4 levels were significantly decreased in mice exposed to OIR (Figure 2C). Localization of FABP4 expression in OIR flat-mounted retinas co-stained with the EC marker BS1 revealed FABP4 to be specifically induced in the neovascular tufts, while normal vessels even closely located to sites of abnormal neovessels showed no FABP4 immunoreactivity (Figure 2D). The specificity of FABP4 staining was confirmed by the absence of staining in OIR retina from FABP4^−/−^ animals. Similarly, confocal analysis of FABP4 expression in OIR cryosections demonstrated its specific expression in pathological neovascular tufts (Figure 3A). Interestingly, FABP4 was not detected in Iba-1 positive microglia/macrophages cells associated with the neovascular structures (Figure 3A) but was clearly detected in the endothelial component of the pathological tufts as demonstrated by its colocalization with the endothelial cell-specific marker endomucin (EMCN) (Figure 3B).

### Genetic deficiency of FABP4 is protective against pathological neovascularization in OIR

**Figure 4:**
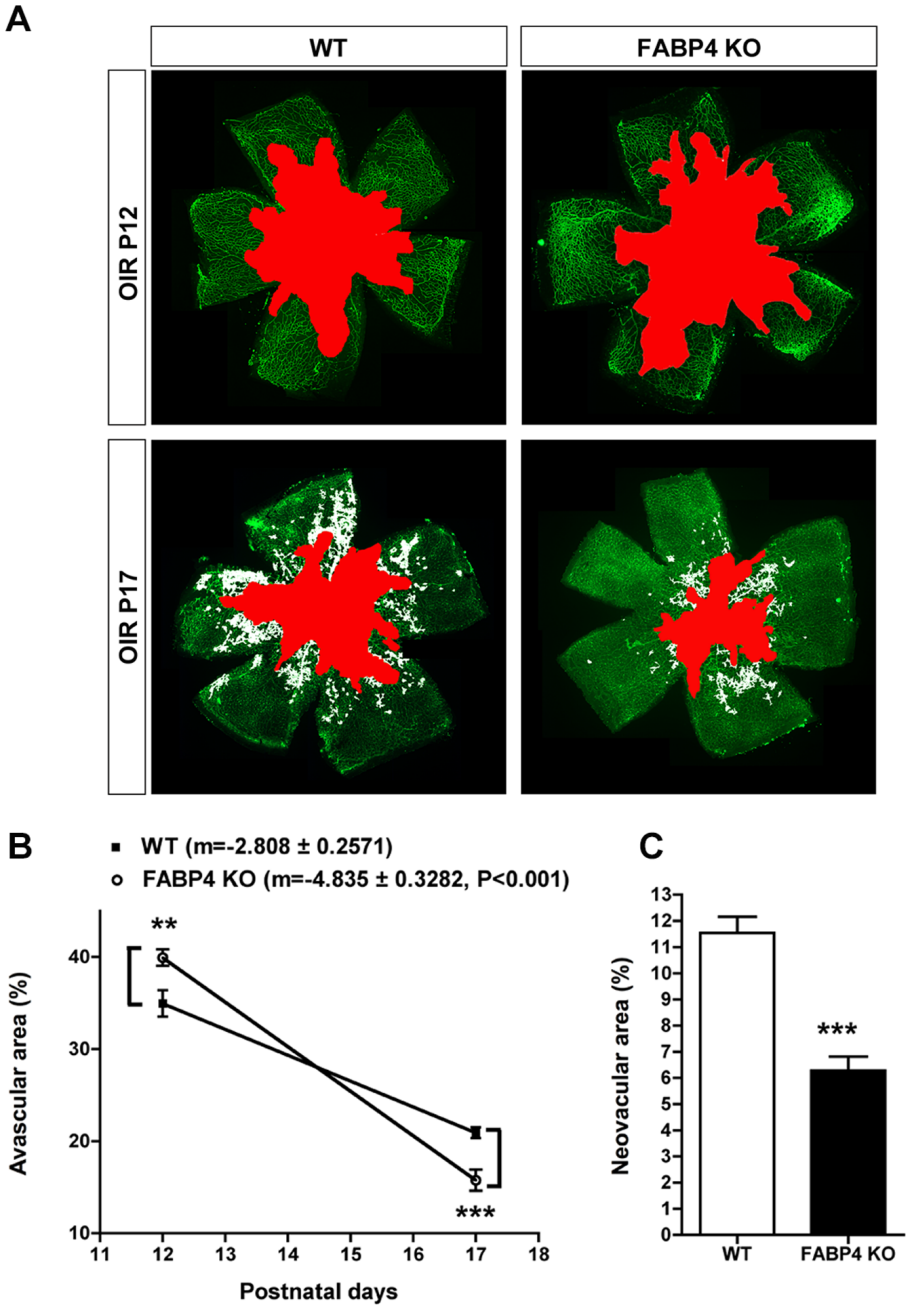
FABP4 deficiency is protective against OIR. (A) Representative FITC-BS1-stained retina flat-mounts from P12 and P17 WT and FABP4^−/−^ mice exposed to hyperoxia. The vaso-obliterated areas are marked in red and the neovascular areas are marked in white. (B) Quantification of the avascular areas shows increased central vaso-obliteration in FABP4^−/−^ retina at P12, which is decreased at P17 (mean ± SEM, n = 7–12 for P12 and n = 15–20 for P17), indicating improved physiological revascularization of avascular areas as indicated by increased slope value (m). (C) Quantification of neovascular area demonstrated a robust inhibition of pathological neovascularization at P17 (mean ± SEM, n = 15–20).

In light of the role of FABP4 in VEGF-dependent signaling in EC and its specific induction in the neovasculature, we next investigated whether FABP4 could regulate pathological vascular proliferation in the OIR model. As the level of angiogenic response in the OIR model is tightly regulated by the extent of the retinal ischemia induced by vaso-obliteration secondary to the hyperoxic treatment[25], we quantified the avascular areas at P12, time of maximal capillary drop-out, and at P17, time of maximal angiogenic response. Lack of FABP4 was associated with a robust reduction in the areas of pathological neovascularization at P17 when compared to WT controls (Figure 4A and C). Interestingly, FABP4^−/−^ mice displayed a significant increase in percentage of vaso-obliteration, when compared to WT mice (Figure 3A and B). There was also a reduction in the central avascular area at P17 in FABP4^−/−^ retinas. Taken together these results suggest that FABP4 deficiency was not only associated with a reduction in pathological neovascularization but also led to a significant improvement in physiological revascularization of the avascular retinal tissues as demonstrated by the significantly increased slope of revascularization between P12 and P17 (Figure 4B).

### Protective effect of FABP4 deficiency in OIR is not associated with altered recruitment of macrophages/microglia

**Figure 5:**
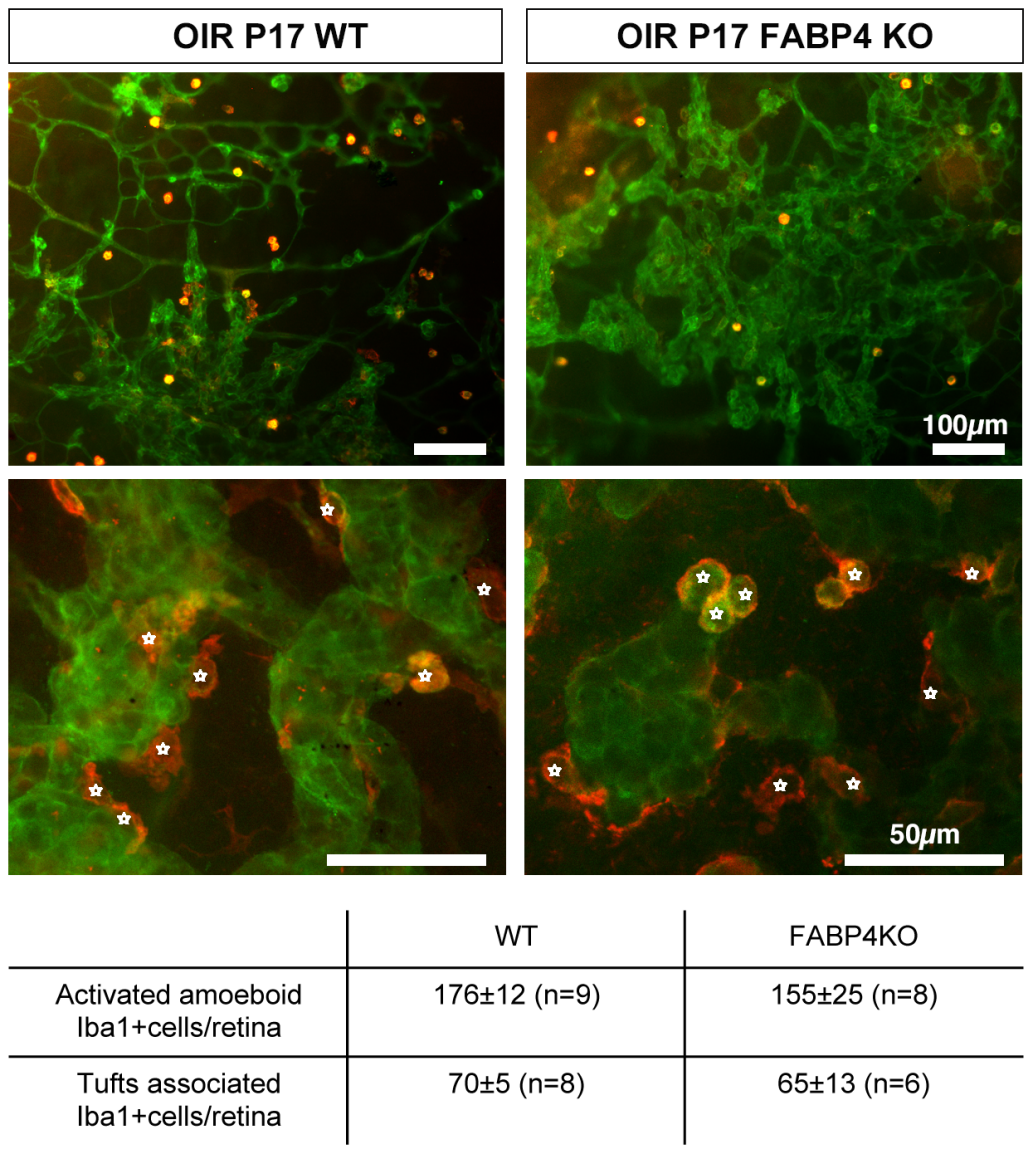
Protective effect of FABP4 deficiency in OIR is not associated with changes in macrophage/microglia recruitment. Activated and neovascular tuft-associated Iba1 positive (red) cells were quantified on FITC-BS1-stained (green) retina flat-mounts of P17 WT and FABP4^−/−^ OIR mice. There were no significant differences in the number of macrophages/microglia recruited to hypoxic retina or those associated with the neovessels (mean ± SEM, n = 9–10).

In the OIR model, recruitment and activation of macrophages are critical components of the pathological angiogenic process as macrophages can secrete angiogenic cytokines and growth factors[26], [27]. Increased secretion of VEGFA and other pro-inflammatory cytokines, such as SDF-1, by the hypoxic retinal tissue leads to robust recruitment and activation of macrophages/microglia as indicated by the appearance of numerous rounded, amoeboid cells intensely stained with the specific marker Iba-1 [27](Figure 5). To determine whether changes in macrophage infiltration could contribute to the reduced retinal neovascularization observed in FABP4^−/−^ animals, we quantified the number of activated macrophages/microglia in P17 retinas. However, there were no differences in the number of activated macrophage per retina or the number of macrophages directly in contact with neovessels in WT versus FABP4^−/−^ mice (Figure 5). These results suggest that, in the OIR model, the protective effect of FABP4 deficiency is not due to reduced macrophage/microglia recruitment or association to the neovascular tufts.

### Protective effect of FABP4 deficiency in OIR is associated with reduced EC proliferation

**Figure 6:**
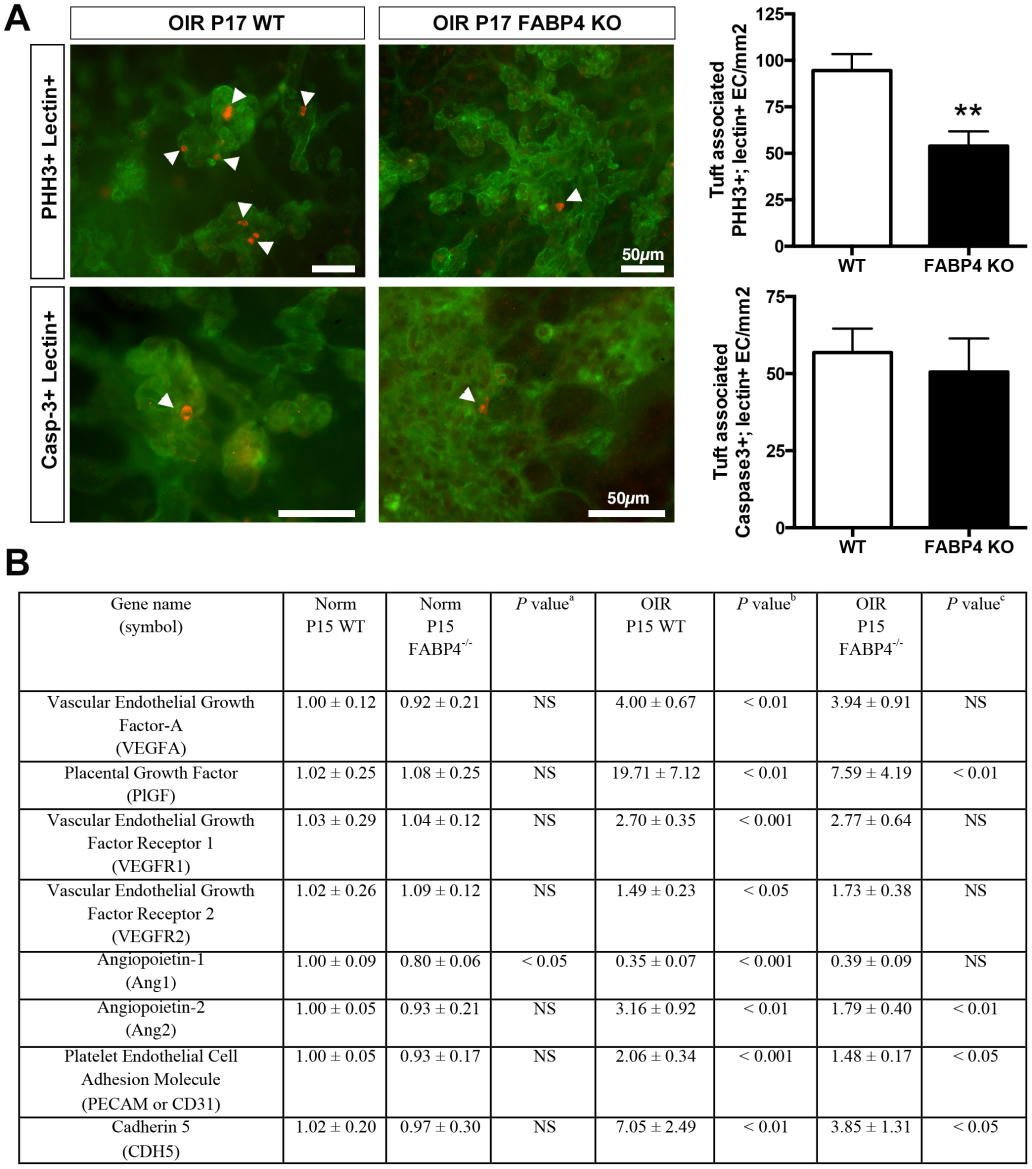
FABP4 deficiency is associated with reduced proliferation of neovascular tufts and differential expression of angiogenesis-related genes. (A) Tufts associated PHH3 positive (red) or activated Caspase-3 positive (red) cells were quantified on FITC-BS1-stained (green) retina flat-mounts of P17 WT and FABP4^−/−^ OIR mice. FABP4 deficiency led to a robust reduction in the number of proliferating ECs in neovascular tufts. No significant difference in the number of apoptotic neovascular EC was observed (mean ± SEM, n  = 4–6). (B) Changes in mRNA levels of selected genes in retinas from P15 WT and FABP4^−/−^ OIR or control mice. The relative steady-state mRNA levels were determined by real-time PCR. Results are presented as mean ± SD based on 4–6 samples from individual mice collected from 2–3 independent litters. NS: not significant, ^a^: Comparison of FABP4^−/−^ control versus WT control; ^b^: comparison of WT OIR versus WT control; ^c^: comparison of FABP4^−/−^ OIR versus WT OIR.

Our previous studies demonstrated that FABP4 promotes proliferation and survival of ECs *in vitro* [19]–[22]. To determine whether protective effect of FABP4 deficiency is associated with reduced EC proliferation or survival, we assessed the index of proliferation and apoptosis in neovascular tufts stained by BS-1 and PHH-3 as a marker of cell division or activated caspase-3 as a marker of apoptosis (Figure 6A). We observed a robust reduction in the number of dividing ECs in FABP4^−/−^ retinal neovessels, but no change in apoptosis suggesting that FABP4 deficiency reduces the proliferative capacity of ECs without having an effect on their survival in the OIR model.

### FABP4 deficiency modulates the expression of angiogenesis-associated genes in OIR

To begin to gain mechanistic insights into the effect of FABP4 in OIR, we analyzed the expression levels of a select group of genes that are known to play roles in the pathogenesis of OIR by real-time PCR analysis of total RNA isolated from P15 mouse retinas (Figure 6B). As expected, mRNA levels of VEGFA, and its receptors VEGFR1 and VEGFR2 were significantly upregulated in WT OIR and were not affected by FABP4 deficiency. In contrast, we detected a significant alteration in the expression level of Placental Growth Factor (PlGF), a member of the VEGF family that binds VEGFR1, which has been shown to stimulate pathological angiogenesis in multiple tissues including the retina[28]. During OIR, we observed a robust induction of PlGF in WT retinas that was significantly blunted in FABP4^−/−^ samples. We observed a similar pattern of expression with Angiopoietin 2 (Ang2) mRNA levels. While Ang1 levels did not show any significant changes between WT or FABP4^−/−^ OIR samples, Ang2 levels showed a significant increase in WT OIR and a significant decrease in FABP4-KO OIR as compared to WT OIR. There were also significant decreases in the mRNA levels of pan-endothelial cell markers CD31 and cadherin 5 in FABP4^−/−^ OIR as compared to WT OIR.

## Discussion

In this study we found that genetic deficiency of FABP4 provides significant protection against the development of OIR in a mouse model. FABP4^−/−^ retinas demonstrated decreased neovascularization, decreased cell proliferation index, and lower mRNA levels of several genes involved in angiogenic responses as compared to WT retinas in the OIR model. Remarkably, FABP4 expression was localized primarily to the neovascular tufts and was absent from normal retinal blood vessels in OIR samples.

We have previously characterized the expression of FABP4 in the vasculature and reported that FABP4 expression was primarily confined to the microvascular endothelial cells in several normal tissues and organs, excluding the normal cerebral and pulmonary vasculature[19], [21]. In this study, we have extended these previous observations and found that FABP4 is not expressed in the normal retinal vessels, but is detected primarily in the microglia and hyaloid vasculature in the postnatal developing retina. FABP4 mRNA levels increased gradually postnatally, reaching the adult levels by P17. As hyaloid vessels normally regress by this age, increased expression levels of FABP4 in the mature retina can likely be accounted by the increase in microglial cells. Consistent with this notion, in a recent study, FABP4 expression was also identified in F4/80-positive cells, a marker for microglia and macrophages, in the adult mouse retina[29]. In addition to microglial cells, we have noted faint FABP4 immunoreactivity throughout the adult mouse retina in non-vascular cells, which may also contribute to the higher expression levels with advancing age. However, it should be noted that despite the significant increase in FABP4 mRNA levels in the developing retina, FABP4 protein levels remained below the detection limit by immunoblot analysis in whole retinal lysates. Thus, FABP4 is expressed at very low levels in the postnatal normal retina.

In the mouse OIR model, hyperoxia exposure between P7 and P12 results in vaso-obliteration of retinal vessels, whereas normoxia exposure between P12 and P17 results in a relative hypoxic state, thus stimulating release of angiogenic factors, which leads to pathological retinal neovascularization. Both mRNA and protein levels of FABP4 were significantly increased in P17 OIR samples compared to normoxic retina samples. Most notably, FABP4 expression was confined to the neovascular tufts and was absent from adjacent normal retinal vessels. Within the pathological neovessels, FABP4 appears to be specifically induced in ECs as it co-localized with the EC markers, EMCN and BS1. This pattern of increased FABP4 expression in OIR closely parallels that of the key angiogenic factor VEGF and is in accordance with our previous studies, which demonstrated induction of FABP4 by VEGF/VEGFR2 pathway in ECs[19]. Since FABP4 appears to be expressed primarily in areas exposed to high levels of VEGF in OIR, FABP4 deficiency or inhibition is unlikely to affect the maintenance or stability of normal retinal vessels as suggested by the normal development of retinal vasculature in FABP4^−/−^ mice. Moreover, the reduction of pathological neovascularization observed in FABP4^−/−^ retina was associated with a significant improvement of the physiological revascularization of hypoxic areas, further supporting the concept that FABP4 deficiency does not have an adverse effect on growth of normal retinal vasculature.

Consistent with our previous studies, decreased neovascularization in FABP4^−/−^ mice was associated with reduced proliferation of ECs, thus implying a role for FABP4 in regulation of angiogenic responses of ECs in OIR. Analysis of a panel of genes that regulate angiogenic responses provided further mechanistic insights into downstream effects of FABP4 in OIR. For example, we found significant decreases in PlGF and Ang2 mRNA levels in FABP4^−/−^ P15 retinas as compared to WT retinas. Although the role of PlGF, a ligand exclusive to VEGFR1, in angiogenic pathologies is controversial, current literature clearly implicates PlGF as an important contributor to retinal neovascularization. PlGF is expressed by several cell type including microvascular ECs [30] and has been shown to stimulate proliferation and migration of retinal ECs in vitro[31]. Sustained intraocular delivery of PlGF led to vascular leakage and anomalies similar to early diabetic retinopathy[32]. Moreover, PlGF deficiency caused a similar effect to that of FABP4 by reducing pathological neovascularization in OIR without affecting normal retinal vascular development[28], [33]. Destabilizing of blood vessels is one of the initial essential processes during angiogenesis and is also regulated by another key mediator, Ang 2, which is also expressed at significantly reduced levels in FABP4^−/−^ retinas[34], [35]. Similar to our observations with FABP4^−/−^ mice, Ang2 hemi-deficiency is associated with reduced neovascularization and avascular area in the OIR model[36]. As both PlGF and Ang-2 are secreted by activated ECs, the angiogenic effect of FABP4 could be in part due to its modulation of EC autocrine and/or paracrine signaling. Further studies will be required to delineate how FABP4 deficiency leads to decreased expression of these two critical mediators and the exact role of these modifications in FABP4-related effects in OIR.

FABP4 has recently been identified as a novel adipokine with potentially important systemic effects[37]. Exogenous administration of FABP4 was able to induce smooth muscle cell proliferation and chemokinesis *in vitro* [38]. Therefore we considered the possibility that circulating FABP4 could contribute to pathological retinal neovascularization. Interestingly, we observed a robust decrease in serum FABP4 levels in P17 pups exposed to OIR. While postnatal high oxygen is not associated with gross changes in body weight and organs function (beside the retina), a potential deleterious effect of hyperoxia exposure on postnatal adipogenesis and/or adipokines secretion cannot be excluded. This observation undermines the potential contribution of circulating FABP4 to retinal pathological neovascularization. Further studies will be necessary to better determine the mechanisms of secretion and systemic and/or paracrine actions of FABP4.

During the progression of OIR, retinal hypoxia may initiate inflammation by direct activation of microglia[39]. In an *in vitro* study, FABP4-deficient macrophages were shown to exhibit decreased inflammatory function with reduced production of inflammatory cytokines, such as IL-1β, TNF-α, and MCP-1, in association with suppressed activity of NF-κB [40]. These previous observations prompted us to consider the possibility that a reduced inflammatory response in the FABP4^−/−^ retina, mediated by either decreased accumulation or activation of microglia could account for the attenuated neovascular responses. However, we did not detect FABP4 expression in microglia/macrophages associated to the neovascular tufts in the retina. Furthermore, we did not detect any differences in the number of activated microglial cells/macrophages between FABP4^−/−^ and WT retinas. Finally, gene expression analysis of pro-inflammatory markers such as TNF-αανδ Arginase-1 showed no significant differential expression between FABP4^−/−^ and WT OIR retinas (data not shown). However, the lack of a conditional FABP4^−/−^ mouse line has hindered our ability to directly address the contribution of macrophage- versus EC-FABP4 in this study.

In summary, our data demonstrate that FABP4 deficiency provides protection in OIR in a mouse model. Lack of FABP4 expression in normal retinal vascular ECs makes FABP4 an attractive target for proliferative retinopathies, including retinopathy of prematurity and diabetic retinopathy.

## Materials And Methods

### Animals

Oxygen-induced retinopathy was induced by placing postnatal (P) 7 mice in 75% ± 2% oxygen for 5 consecutive days, as previously described[24]. FABP4^−/−^ mice on C57BL/6 background[15] and FABP4^+/+^ littermates or C57Bl/6 mice from the Jackson Laboratories were used in all experiments.

### Ethics Statement

The Harvard Medical Area Standing Committee on Animals approved all animal procedures.

### Real-time PCR

Total RNA was isolated from tissue and cells using RNA-bee solution (IsoText Diagnostic). RNA was reverse-transcribed using iScript (Biorad) or the High Capacity cDNA Reverse Transcription Kit (Applied Biosystems). qPCR reactions were performed using the SYBR Green Master mix and the ABI Prism 9700 Sequence Detection System (Applied Biosystems) according to the manufacturer's instructions. Accurate gene expression was calculated relative to the housekeeping genes, HPRT1 and PPIA, according to the ΔΔCt method. Analyzed genes and corresponding primer sequences are listed in Table 1. Primer pair for Vascular Endothelial Growth Factor Receptor 2 (VEGFR2) was purchased as validated premix from SABiosciences (Qiagen).

**Table 1:** Real-time PCR primer sequences.

Gene name (symbol)	Forward primer (5′-3′)	Reverse primer (5′-3′)
Hypoxanthine Phosphoribosyltransferase 1 (HPRT1)	TCAGTCAACGGGGGACATAAA	GGGGCTGTACTGCTTAACCAG
Peptidylprolyl Isomerase A (PPIA)	GAGCTGTTTGCAGACAAAGTTC	CCCTGGCACATGAATCCTGG
Fatty Acid Binding Protein 4 (FABP4)	TCACCATCCGGTCAGAGAGTA	GCCATCTAGGGTTATGATGCTC
Vascular Endothelial Growth Factor-A (VEGFA)	GCACATAGAGAGAATGAGCTTCC	CTCCGCTCTGAACAAGGCT
Placental Growth Factor (PlGF)	CTGTGTGCCGATAAAGACAGC	GGTTCCTCAGTCTGTGAGTTTC
Vascular Endothelial Growth Factor Receptor 1 (VEGFR1)	CTCAGGGTCGAAGTTAAAAGTGC	TTGCCTGTTATCCCTCCCACA
Angiopoietin-1 (Ang1)	CCATGCTTGAGATAGGAACCAG	TTCAAGTCGGGATGTTTGATTT
Angiopoietin-2 (Ang2)	GCTCCTTCATGGACTGTAGCTG	AGCAGATTTTGGATCAGACCAG
Platelet Endothelial Cell Adhesion Molecule (PECAM-1 or CD31)	GAGGAAAGCCAAGGCCAAACAG	TGGCTTCCACACTAGGCTCAGA
Cadherin 5 (CDH5)	TTTGCCCTGAAGAACGAGGACA	ATGCTCCCGATTAAACTGCCCA

### Western blot

WT and FABP4 eyes were dissected under a dissecting microscope, the retinas were isolated and homogenized in Lysis Buffer (Cell Signaling). Each sample corresponded to one individual mouse. Protein concentration was quantified using a BCA assay (Bio-Rad, Hercules, CA). For western-blot analysis, identical protein amount was separated by SDS-page under reducing condition and transferred to Immobilon-P membrane (Millipore, Bedford, MA). Membranes were incubated overnight at 4°C with FABP4 (1∶1000, ab97963 lot#GR27611-7, Abcam) or beta-actin (1∶1000, Santa-Cruz) antibodies. Then the membrane was incubated with corresponding secondary antibodies (1∶10000, Jackson ImmunoResearch Laboratories) in 5% milk, 3% BSA in TBST buffer for 1 hour at room temperature. Signal intensity was determined by densitometry (Quantity One; Bio-Rad, Hercules, CA) and normalized to the amount of beta-actin in each sample.

### Immunohistochemistry

Whole retinas were dissected and immunostained as previously described[9]. Briefly, whole retinas were blocked 2 hours with 1% BSA in PBS containing 0.1 mM CaCl_2_, 0.1 mM MgCl_2_ and 0.1 mM MgCl_2_ and incubated overnight with the appropriate antibody: Alexa 488-labeled or Alexa 594-labeled isolectin *Bandeiraea simplicifolia* 1 (BS1, Invitrogen), anti-Collagen IV (Abcam) anti-FABP4 (Abcam and LS-B4227 lot#25048, LifeSpan BioSciences), anti-phospho histone H3 (ser10) (PHH3, Millipore) or anti-Iba1 (Wako). Primary antibodies are visualized using Cy5-, Cy3-, Alexa 594 or Alexa 488 conjugated secondary antibodies (Jackson ImmunoResearch Laboratories) or using a biotinylated secondary antibody and detected with the Vectastain ABC system (Vector). Vascular outgrowth, avascular and neovascular area were measured on BS1 stained and flat-mounted retinas using Photoshop CS4 as previously described[25]. For confocal analysis, retina flat-mounts were co-immunolabeled with rabbit-raised anti-FABP4 (LifeSpan Bioscience), rat-raised anti-endomucin (EMCN) (Santa Cruz Biotechnology), goat-raised Iba1 (Abcam). Vascular density at P7 was measured on collagen IV stained retina flat-mounts and quantified on 6 fields/retina with each field representing 0.057 mm^2^ located midway between the optic disc and the periphery and away from any main arteries or veins using Image J software (National Institute of Health). Briefly, blood vessels stained for collagen IV were distinguished from background using empirically determined pixel brightness threshold values that included only blood vessels. The vascular area was calculated as the number of pixels having a brightness value higher than the threshold. The mean value was calculated for each mouse. Quantification of Iba1 positive cells was performed on Iba1 and BS1 co-stained flat-mounts using 10 fields/retina at a 20× magnification for total number and 5 fields/retina a 40× magnification for cells associated with neovascular tufts. Quantification of proliferative and apoptotic endothelial cells was performed blindly on flat-mounted retina co-stained for the proliferation marker PHH3 and the endothelial marker BS1, or for the apoptosis marker active caspase-3 and BS1, respectively, using 6 fields/retina at a 40× magnification randomly selected in the mid-peripheral region. The number of PHH3 or active caspase-3 positive endothelial cells per fields was quantified and normalized to the area of neovascular tufts.

### Measurement of FABP4 levels in serum

Non-fasting blood of P17 WT control and OIR pups was collected from the orbital sinus. Serum was prepared by centrifugation of the clotted blood samples for 10 min at 4°C and immediately assayed. Serum FABP4 levels were quantified using a mouse FABP4 ELISA kit (Circulex) following the manufacturer's instructions.

### Statistical analysis

Values are expressed as mean ± SEM (unless specified) and statistical analysis was performed using an unpaired Student t test (***: P<0.001, **: P<0.01, *: P<0.05, ns: P>0.05).

## Supporting Information

Figure S1 **FABP4 expression in ocular tissues.** Immunolocalization of FABP4 in adult (8 weeks old) ocular tissue confirmed the expression of FABP4 in endothelial cells of the periorbital muscles vasculature and limbal vessels. Brown fat was used as positive control and as expected a strong signal was detected in adipocytes. Scale bar is 100 µm.
(TIF)
