# Supplementary figures and images for: Fatty Acid Binding Protein 4 Deficiency Protects against Oxygen-Induced Retinopathy in Mice
Source: PLoS One. 2014 May 6;9(5):e96253. doi: 10.1371/journal.pone.0096253 (PMC4011730; doi:10.1371/journal.pone.0096253)

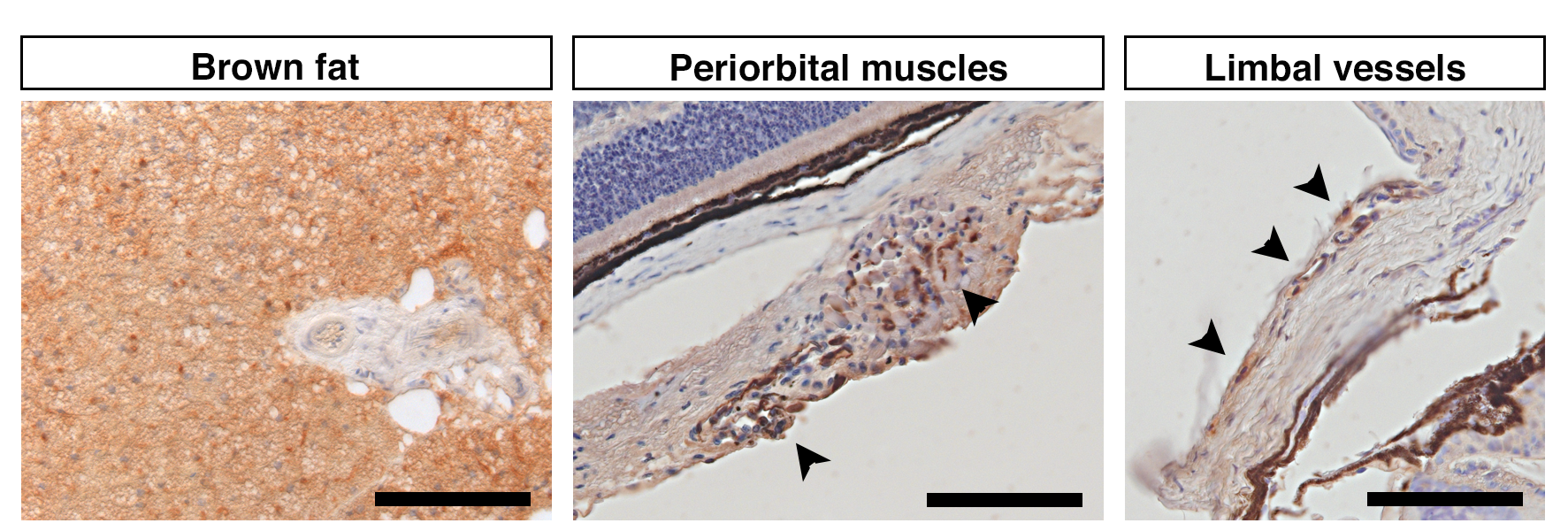

Supplement: Figure S1 — FABP4 expression in ocular tissues. Immunolocalization of FABP4 in adult (8 weeks old) ocular tissue confirmed the expression of FABP4 in endothelial cells of the periorbital muscles vasculature and limbal vessels. Brown fat was used as positive control and as expected a strong signal was detected in adipocytes. Scale bar is 100 µm. (TIF) [file pone.0096253.s001.tif]
